# GPR37 and Related Receptors: Disease Regulation

**DOI:** 10.3390/ijms24076722

**Published:** 2023-04-04

**Authors:** Daniela Marazziti

**Affiliations:** Institute of Biochemistry and Cell Biology, Italian National Research Council, 00015 Monterotondo, Rome, Italy; daniela.marazziti@cnr.it; Tel.: +39-0690091202

The vertebrate G protein-coupled receptors 37 and 37-like 1 (GPR37 and GPR37L1) were discovered more than two decades ago, and they have been implicated in various neurological and neurodegenerative diseases, as well as in inflammatory pain and tumorigenesis. Several aspects of their physiology are still actively investigated, as their actual involvement in many interesting areas of research has been only partially explored.

Both receptors are distinctively expressed in the brain by neuronal and glial cells, and recent studies have also reported their specific expression in peripheral tissues. They have been found to functionally interact with several transmembrane proteins, modulating their intracellular processing and surface expression. Furthermore, GPR37, but not GPR37L1, has a propensity to form cytotoxic intracellular aggregates upon, e.g., overexpression and misfolding.

The proposed interaction of both receptors with prosaposin and derived cytoprotective ligands is still actively debated. Meanwhile, the neuroprotectin D1 bioactive lipid and osteocalcin bone-derived hormone have also been indicated as specific ligands for GPR37, to regulate, respectively, macrophage phagocytosis and myelin homeostasis in the central nervous system.

The aim of the International Journal of Molecular Sciences’ Special Issue entitled “GPR37 and Related Receptors: Disease Regulation” was to stimulate the publication of novel results and perspectives from experts in this field of biomedical research.

The contributed reviews highlight the recently reported roles played by GPR37 and GPR37L1 in some brain diseases, such as stroke, pain and megalencephalic leukoencephalopathy with subcortical cysts (MLC), or analyze in detail types and applications of available mouse mutant strains for both receptors. The research articles present new analysis on previously unexplored aspects of behavioral or epigenetic functions.

The recently discovered role of GPR37 in inflammation and pain is addressed in the review by Zhang et al. [1]. The authors discuss in particular the beneficial or detrimental role of GPR37 in several pathological conditions, ranging from neurological disorders to stroke or cancer. They also focus on GPR37’s regulation of macrophage phenotypes and its involvement in resolving inflammatory pain upon binding to neuroprotectin D1. On the basis of reported data, they conclude that this receptor could be a target for developing novel drugs, aimed at treating certain inflammatory and pain conditions, neurological diseases and infections, such as malaria.

Mouhi et al. [2] review the possible involvement of both receptors in regulating various cellular and molecular processes underlying ischemic stroke, with particular focus on induced systemic inflammatory responses. A critical discussion of both receptors’ proposed ligands and interacting protein partners is also presented. A special section is dedicated to the N-terminal processing of both receptors by metalloprotease cleavage. The authors underscore the importance of characterizing which receptor forms are present in different physiological or pathological conditions and understanding how N-terminal cleavage is actually related to the activation or inactivation of a receptor’s signaling.

Pla-Casillanis et al. [3] discuss the functions of GPR37 and GPR37L1 as part of the interactome of brain glial cell adhesion molecule (GLIALCAM), one of the proteins associated with the MLC vacuolating leukodystrophy, in addition to MLC1. The *MLC1* and *GLIALCAM* genes encode for still incompletely characterized membrane proteins, which are involved in the functional regulation of different ion channels and transporters. MLC1 and GLIALCAM form protein complexes and various experimental investigations suggest that both GPR37 and GPR37L1 could act as negative regulators of such complexes in different glial cell populations.

Several studies of murine mutant models have been instrumental for elucidating both receptors’ functions, and the review by Massimi et al. [4] summarizes the genotypic and phenotypic characteristics of published *Gpr37* and *Gpr37l1* mouse mutant strains, in brain or peripheral tissues. For each receptor gene, several knock-out mutant lines, either constitutive or conditional, are described, as well as two GPR37- and one GPR37L1-expressing transgenic lines. The possibility of using some of the reported strains as specific disease models is extensively discussed, also considering the application of newly available reporter-tagged, constitutive or cell type-specific knock-out mouse strains, on co-isogenic C57BL/6N background.

Behavioral phenotypes of mouse *Gpr37* or *Gpr37l1* knock-out strains have been analyzed by two original research papers [5,6]. GPR37 has been associated with Parkinson’s disease, bipolar and major depressive disorders, and mouse mutant strains have been analyzed for relevant motor and non-motor functions. Veenit et al. [5] investigate emotional and cognitive domains to study how early life stress (ELS) affects, in a gender-specific fashion, the behavior of adult *Gpr37* null mutant mice, based on the hypothesis that genes implicated in neurodegeneration could influence early brain development. The results of a battery of specific tests confirm that ELS has effects on various emotional behaviors of wild-type but not knock-out mice, which showed resilience towards ELS in a gender- and context-dependent manner, confirming and extending previously reported data. 

Gender- and age-dependent emotional and cognitive behavior has also been studied in *Gpr37l1* null mutant mice [6], upon specific application of comprehensive test panels. Significant variations in the levels of proteins known to modulate anxiety, depression, and memory, have also been measured and reported. GPR37L1 is found involved in mediating aversive memory and its specific deletion in dorsal hippocampal astrocytes markedly decrease the retention latency in passive avoidance tests. In general, the lack of GPR37L1 influences behavioral and biochemical readouts in age- and gender and age-specific manners.

Armando et al. [7] demonstrate that GPR37L1 is functionally localized at the nuclear membrane and specifically modulates renal sodium transport, through epigenetic regulation. The authors characterize the protein complexes associated with the receptor, in human renal proximal tubule cells and identify several functional partners involved in the control of nuclear membrane trafficking. They also show that GPR37L1 contains a nuclear membrane localization signal and can enhance the transcription of the sodium/proton exchanger isoform 3 (*NHE3*) gene via the mTOR phosphorylation pathway, upon methylation of the *NHE3* promoter region.

All contributions to this Special Issue clearly underscore the complexity of GPR37 and GPR37L1 receptor’s functions in many organs, tissues and cell types and evidence the need for further research on many unresolved issues and their implications for human health.
